# Are Inflammatory Markers and Periodontitis Effective in Predicting Miscarriage?

**DOI:** 10.3390/healthcare13131565

**Published:** 2025-06-30

**Authors:** Isa Temur, Selcen Ozcan Bulut, Safak Necati Dönertas, Aycan Dal Dönertas, Katibe Tugce Temur, Guldane Magat

**Affiliations:** 1 Department of Obstetrics and Gynecology, Faculty of Medicine, Niğde Ömer Halisdemir University, 51240 Niğde, Türkiye; t.isatemur@gmail.com; 2 Department of Periodontology, Faculty of Dentistry, Niğde Ömer Halisdemir University, 51240 Niğde, Türkiye; selcen_ozcan@hotmail.com (S.O.B.); safak_necati@hotmail.com (S.N.D.); 3 Department of Pedodontics, Faculty of Dentistry, Niğde Ömer Halisdemir University, 51240 Niğde, Türkiye; aycandal@outlook.com; 4 Department of Oral and Maxillofacial Radiology, Faculty of Dentistry, Niğde Ömer Halisdemir University, 51240 Niğde, Türkiye; 5 Department of Oral and Maxillofacial Radiology, Faculty of Dentistry, Necmettin Erbakan University, 42090 Konya, Türkiye; gul_dent@hotmail.com

**Keywords:** inflammation markers, periodontal health, miscarriage

## Abstract

**Background/Objectives:** Miscarriage is a common pregnancy complication that significantly impacts individuals’ health due to its physical and psychological effects. This study aimed to investigate the association between periodontal health and hematological parameters in women who experienced miscarriage before the 20th week of gestation, and to assess the potential predictive value of these parameters for miscarriage risk by comparing them with those of women with an uncomplicated pregnancy course. **Methods:** This study was a prospective case–control and cross-sectional study. It included a total of 82 participants, comprising 41 women with miscarriage and 41 healthy pregnant controls. The periodontal examinations included measurements of the Gingival Index (GI), Plaque Index (PI), Probing Depth (PD), Clinical Attachment Loss (CAL), and Simplified Calculus Index (SCI). Additionally, complete blood counts (CBCs) were obtained from all participants. Appropriate statistical analyses, including non-parametric, correlation, logistic regression, and ROC analyses, were conducted, with the significance level set at *p* < 0.05. **Results:** The primary outcome measure was CAL as an indicator of periodontal disease severity and its association with miscarriage risk. Additional outcomes included Plateletcrit (PCT), the Platelet Count (PLT), and the Neutrophil-to-Lymphocyte Ratio (NLR) to evaluate systemic inflammatory responses and their correlations with periodontal parameters. CAL was significantly elevated in the miscarriage group (*p* < 0.001) and emerged as the strongest predictor of miscarriage risk (OR = 0.0537, *p* < 0.001, AUC = 0.8691). PCT was significantly higher in the miscarriage group (*p* = 0.017) and positively correlated with the GI (*p* = 0.041), suggesting a link between systemic inflammation and periodontal health. **Conclusions:** Considering this study’s limitations, CAL was the strongest predictor of miscarriage, while PLT and PCT had some discriminative power. Collaboration between obstetricians and dentists can facilitate early diagnosis and intervention by promoting routine oral health check-ups before and during pregnancy. Additionally, integrating oral health assessments into prenatal care and developing public health policies could enhance access to dental services during both preconception and pregnancy periods.

## 1. Introduction

Miscarriage is a prevalent complication in human pregnancies, with research indicating that approximately 10–20% of clinically recognized pregnancies culminate in pregnancy loss [1,2]. Miscarriage is associated with physical complications such as bleeding and infection, as well as psychological effects such as anxiety, depression, and post-traumatic stress disorder. Recurrent pregnancy loss is a well-established predictor of adverse obstetric outcomes in subsequent pregnancies, including preterm birth, fetal growth restriction, placental abruption, and stillbirth. Moreover, emerging evidence suggests that miscarriage may serve as an early indicator of long-term health risks, such as cardiovascular disease [3]. The risk factors associated with miscarriage are multifaceted, encompassing psychosocial stress, certain medications, systemic infections, active smoking during pregnancy, and reproductive history, including a family history of miscarriage [4,5]. Despite extensive research, considerable uncertainties persist regarding its underlying causes, recurrence risk, and the effectiveness of preventive interventions. Additionally, although specialized clinics offer care for couples experiencing miscarriage, significant disparities exist in the management approaches and treatment strategies across different healthcare settings [6]. In recent years, the systemic implications of periodontitis have drawn increasing attention, particularly its potential role in adverse pregnancy outcomes. Periodontitis, a chronic inflammatory condition initiated by subgingival biofilm, has been linked to systemic disorders such as cardiovascular diseases and diabetes [7,8]. Several studies have suggested that periodontitis may contribute to pregnancy complications, including preterm birth and pregnancy loss [9]. However, research specifically investigating its association with miscarriage remains limited [10]. Its potential mechanisms include systemic dissemination of periodontal pathogens, the elevation of inflammatory mediators that may induce uterine contractions, and immune dysregulation, particularly in conditions such as antiphospholipid syndrome [11]. Understanding the interplay between periodontal health and miscarriage is critical, especially as systemic inflammatory parameters from complete blood counts (CBCs) offer a cost-effective and accessible means for assessing health in pregnant females [9,12].

In the limited number of studies conducted in this field, the association between periodontitis and spontaneous miscarriage has been evaluated using various methodologies. Case–control studies have generally found periodontitis to be more prevalent in women who have experienced miscarriage, whereas a prospective cohort study based on self-reported data did not reveal a significant association [13,14]. Nevertheless, a history of tooth mobility, which is considered an indicator of periodontitis severity, has been reported to show a weak but positive association with miscarriage risk [14]. A recent meta-analysis indicated a potentially significant association between periodontal disease and pregnancy loss, particularly in cohort studies; however, it also emphasized the conflicting nature of the current evidence and the need for further high-quality research in this area [15].

This study distinguishes itself from many previous investigations by simultaneously assessing periodontal status and hematological parameters in pregnancies that ended in miscarriage. Specifically, the evaluation of both periodontal findings and cost-effective, readily accessible hematological markers such as complete blood counts during early pregnancy losses represents a novel approach aimed at uncovering the predictive potential of these parameters in relation to miscarriage risk.

This study investigates the potential link between periodontal status and hematological markers in women who experienced miscarriage prior to the 20th week of gestation, aiming to determine whether these parameters can serve as predictors of miscarriage risk by conducting a comparative analysis with women who had a normal pregnancy progression.

## 2. Materials and Methods

### 2.1. Ethical Approval for the Study

This study received approval from the Non-Interventional Ethics Committee of Nigde Ömer Halisdemir University Faculty of Medicine (Date: 28 July 2022, Decision No.: 2022/71) and was conducted in full compliance with all versions of the Declaration of Helsinki.

### 2.2. Study Design and Participant Groups

This study was conducted between September 2022 and 2023 at the Department of Obstetrics and Gynecology, Niğde Ömer Halisdemir University. Women with a history of miscarriage were included in the case group, while healthy pregnant women constituted the control group. Only individuals between the ages of 18 and 35 were enrolled in this study. Written informed consent was obtained from all participants prior to their inclusion.

The case group consisted of women diagnosed with miscarriage before the 20th gestational week. Gestational age was determined using the first day of the last menstrual period (LMP), as reported by the mother at her initial prenatal appointment. However, in cases where the LMP was uncertain or not definitive, gestational age was confirmed through ultrasonographic measurements.

The control group consisted of women who underwent an oral examination and had blood parameters assessed before the 20th week of gestation, who were subsequently followed and confirmed to have completed a healthy pregnancy and delivered at term.

In this study, a power analysis was conducted using Fisher’s exact test to evaluate inequality between the proportions of two independent groups. The analysis was planned with a 5% significance level (α = 0.05) and 95% test power (1 − β = 0.95), determining that a total of 66 participants—33 in each group—would be sufficient to achieve adequate power [13]. Accordingly, a total of 82 participants were included in this study, comprising 41 cases and 41 controls, resulting in a larger sample size than the minimum required by the power analysis.

### 2.3. Inclusion Criteria

- Females aged 18–35 years.
- Case group: Females with no prior history of miscarriage who experienced a miscarriage before the 20th gestational week.
- Control group: Females with an ongoing, healthy, uncomplicated pregnancy and no history of miscarriage.
- All participants received care at the same hospital and were assessed to have similar socioeconomic characteristics.

### 2.4. Exclusion Criteria

- The presence of systemic diseases (e.g., diabetes mellitus, bleeding disorders, autoimmune diseases, or other chronic conditions) that could affect study outcomes.
- A history of pregnancy-related complications (e.g., preeclampsia, gestational diabetes) in previous pregnancies.
- The use of antibiotics or antimicrobial mouthwashes within the past 3 months.
- Periodontal treatment received within the past 6 months.
- Fewer than 20 natural teeth, compromising reliable periodontal assessment.
- Regular smoking or alcohol consumption.
- The presence of uterine anomalies.
- A known history of fetal chromosomal abnormalities.

### 2.5. Blood Parameters

CBC parameters were analyzed for all participating at the Hemogram Central Laboratory of Niğde Ömer Halisdemir University Training and Research Hospital using an automated hematology analyzer (Sysmex Corporation, Kobe, Japan).

The analysis included the Platelet Count (PLT), Mean Platelet Volume (MPV), Mean Platelet Volume-to-Platelet Ratio (MPV/PLT), Monocyte-to-Lymphocyte Ratio (MLR), Platelet-to-Lymphocyte Ratio (PLR), Neutrophil-to-Lymphocyte Ratio (NLR), White Blood Cell Count (WBC), Plateletcrit (PCT), and Neutrophil Count (NC). Venous blood samples were collected in the morning (between 08:00 and 10:00 AM) to account for circadian variations in hematological parameters. All participants were instructed to rest in a seated position for at least 5 min before blood collection to prevent hemoconcentration effects. A tourniquet was applied for less than 1 min to avoid artificial alterations in blood cell counts. Blood samples were drawn into ethylenediaminetetraacetic acid (EDTA) tubes (Becton Dickinson, BD Vacutainer^®^, Franklin Lakes, NJ, USA) and immediately transported to the laboratory for analysis. To ensure data accuracy and minimize pre-analytical variability, all samples were analyzed within 2–4 h of collection to prevent platelet aggregation and cellular degradation.

### 2.6. Periodontal Examination

Oral examinations were conducted during routine prenatal visits by two independent periodontologists (SOB and SND), each with more than five years of clinical experience, who were blinded to the participants’ systemic and obstetric data. The assessments were performed at the time of miscarriage diagnosis for the case group and during the corresponding gestational period for the control group. All examinations were performed while the patients were seated on an examination chair located in the area where the pregnancy diagnosis had been made. During the examinations, the patients’ heads were stabilized, and a portable light source was used to ensure adequate illumination. All procedures were conducted following the same standardized method.

For each participant, periodontal parameters, including the Gingival Index (GI), Plaque Index (PI), Probing Pocket Depth (PD), Clinical Attachment Loss (CAL), and Simplified Calculus Index (SCI), were assessed using a periodontal probe and mouth mirror (Hu-Friedy, Chicago, IL, USA). PI, GI, and PD measurements were recorded from four sites per tooth to enhance diagnostic accuracy and reproducibility [16].

### 2.7. Inspection Measurement Reliability

To assess the reliability and consistency of periodontal measurements, the intraclass correlation coefficient (ICC) was calculated to determine inter-reviewer reliability by comparing two separate datasets obtained from measurements performed by the periodontologists, SOB and SND.

### 2.8. Statistical Analysis

In this study, statistical analyses were performed using SPSS (Statistical Package for the Social Sciences) software, Version 26.0 (IBM Corp., Armonk, NY, USA). Descriptive statistics were used to evaluate age and baseline variables across the groups, while the Mann–Whitney U test was applied to examine differences between the case and control groups. Relationships between blood parameters and oral indices were assessed using Spearman’s correlation analysis. A hierarchical binary logistic regression analysis was conducted to identify factors determining miscarriage risk, with effects reported using odds ratios (ORs) and *p*-values. To evaluate the discriminative power of parameters in distinguishing case groups, a Receiver Operating Characteristic (ROC) analysis was performed, and the Area Under the Curve (AUC) values were calculated. Additionally, the chi-square test was used to assess relationships between categorical variables. A *p*-value of 0.05 was used to represent statistical significance.

## 3. Results

A total of 82 individuals participated in this study (41 cases and 41 controls). There was no significant age difference between the groups (cases: 27.15 ± 5.09 years; controls: 26.15 ± 4.17 years; *p* > 0.050).

Significant differences were noted in blood parameters (Table 1). The PLT (*p* = 0.004), the MLR (*p* = 0.004), and PCT (*p* = 0.017) were higher in the case group, while the MPV/PLT (*p* = 0.001), NLR (*p* < 0.001), WBC (*p* = 0.002), MPV (*p* = 0.031), and NC (*p* < 0.001) were higher in controls. The PLR showed no significant difference between the groups (*p* > 0.050).

Among the oral indices, significant differences were observed only in the CAL parameter (Table 2). CAL was significantly higher in the case group (2.02 ± 1.99) than in the control group (0.71 ± 0.00, *p* < 0.001). Other parameters, such as the pocket depth, GI, PI, and SCI, did not show statistically significant differences between the groups (*p* > 0.05) (Table 2).

In Spearman’s correlation analyses, PCT correlated positively with the GI in the case group (r = 0.320, *p* = 0.041) (Table 3), while the PLR showed a negative correlation with CAL in controls (r = −0.352, *p* = 0.024) (Table 4). No other significant correlations were observed in the case and control groups (*p* > 0.05).

The hierarchical logistic regression identified key miscarriage risk factors (Table 5). In Model 1, CAL significantly increased risk (*p* < 0.001, OR = 0.054). In Model 2, the PLT (*p* = 0.009, OR = 0.9997) and the NLR (*p* = 0.036, OR = 0.044) reduced risk, while PCT (*p* = 0.033, OR = 1.19029 × 10^87^) and the NC (*p* = 0.043, OR = 10.527) increased risk.

**Figure 1:**
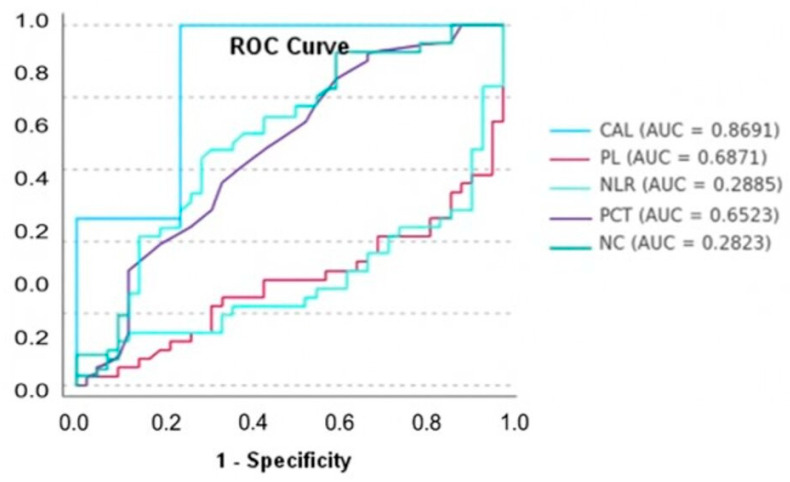
ROC curve analysis for predicting miscarriage risk (AUC: Area Under the Curve; CAL: Clinical Attachment Loss; PLT: Platelet count; NLR: Neutrophil-to-Lymphocyte Ratio; PCT: Plateletcrit; NC: Neutrophil Count).

The ROC analysis showed that CAL had the strongest discriminatory power (AUC = 0.869), followed by PLT (AUC = 0.687) and PCT (AUC = 0.652). The NLR and NC had poor performance (AUC < 0.300) (Table 6, Figure 1).

The inter-reviewer reliability was assessed to evaluate the consistency and agreement between the reviewers. The intraclass correlation coefficients (ICCs) calculated for all quantitative variables ranged between 0.80 and 0.90, demonstrating excellent reliability in line with established standards for reproducibility.

## 4. Discussion

Miscarriage is a common pregnancy complication that significantly impacts individuals’ health through both physical and psychological effects. While the influence of periodontitis on pregnancy complications is a critical subject, there are notable gaps in knowledge and research regarding its genetic and pathogenetic effects, particularly on miscarriage. To the best of our knowledge, this is the first study to investigate the potential systemic effects of periodontal health in women with a history of miscarriage by assessing its influence on inflammatory blood markers derived from a CBC.

In this study, PLTs, MLRs, and PCT levels were significantly higher in women who experienced miscarriage. In contrast, the MPV/PLT, NLR, WBC, MPV, and NC were significantly higher in the control group. However, no significant difference was observed between the groups in terms of the PLR. These findings suggest that certain hematological parameters may be associated with pregnancy outcomes and could reflect distinct inflammatory response profiles associated with miscarriage. While some previous studies support these findings, others have reported conflicting results.

For instance, Soysal et al. [17] reported significantly elevated systemic immune-inflammation index (SII) values, NLRs, MLRs, red cell distribution widths (RDWs), and PLRs in patients diagnosed with threatened abortion. These findings support the hypothesis that multiple inflammatory pathways may play a role in the pathogenesis of threatened abortion. Furthermore, their study suggested that the SII may serve as a more robust predictor of inflammation and miscarriage risk during ongoing pregnancies compared to markers such as the NLR and PLR.

While Akın et al. [18] also reported significantly higher MPVs, PCT levels, and PLRs in women who experienced miscarriage, only their PCT findings are consistent with those of the current study. In contrast, MPVs were higher in the control group in the present analysis. Notably, both studies found differences in the PLR to be statistically nonsignificant between groups, suggesting that the PLR may not be a reliable marker for predicting miscarriage risk.

Similarly, Gül et al. [19] found that women with early pregnancy loss had elevated MPVs, platelet distribution widths (PDWs), PLRs, PLTs, eosinophil-to-lymphocyte ratios (ELRs), and eosinophil-to-neutrophil ratios (ENRs), whereas the control group showed higher WBCs and higher hemoglobin, neutrophil, and lymphocyte levels. The observation of elevated WBCs and NCs in the control group aligns with the current findings. However, the increased MPVs in the miscarriage group reported by Gül et al. are inconsistent with those observed in this study.

These discrepancies may be attributed to the diversity of hematological parameters assessed and the methodological differences across studies, which make direct comparisons difficult. Additionally, variations in sample sizes, diagnostic criteria, measurement timings, and demographic characteristics of the study populations may contribute to the inconsistencies observed in the literature.

One of the most notable findings of this study is the statistically significant difference in CAL, a key clinical indicator reflecting the health of periodontal tissues and the extent of damage to the supporting structures of the teeth, between the case and control groups (2.02 ± 1.99 vs. 0.71 ± 0.00, *p* < 0.001). The higher CAL values observed in the case group suggest greater periodontal tissue damage and a marked deterioration in periodontal health among these individuals. The significant elevation in CAL among those with a history of miscarriage further underscores the potential implications of periodontal disease on reproductive health. Previous research has suggested a link between periodontal disease and adverse pregnancy outcomes, reinforcing the idea that oral health is an integral component of overall health during pregnancy [11]. The finding that increased CAL was observed in the case group raises concerns that periodontal disease may contribute to the risk of miscarriage, as the chronic inflammation associated with periodontal disease could disrupt the delicate immunological and vascular balance necessary for maintaining pregnancy [20]. However, in contrast to CAL, no significant differences were found between the groups for other periodontal indices, including the PD, GI, PI, and SCI (*p* > 0.05). This finding aligns with the results reported by Harris et al. [21], which suggested that periodontal disease, characterized by increased pocket depth or CAL, is associated with an elevated risk of late miscarriage or stillbirth. Similarly, in a study conducted by Bond et al. [14], while the diagnosis and treatment of periodontitis did not have a significant relationship with miscarriage, a history of tooth mobility was positively associated with miscarriage. However, this association was not statistically significant. Conversely, a retrospective cohort study demonstrated that periodontal disease significantly increased the risk of miscarriage, aligning with findings from a meta-analysis that identified a strong association between periodontal disease and adverse pregnancy outcomes. The meta-analysis, which included 17 case–control studies with a total of 10,148 patients, reported estimated odds ratios of 1.78 (95% CI: 1.58, 2.01) for preterm birth, 1.82 (95% CI: 1.51, 2.20) for low birth weight, and 3.00 (95% CI: 1.93, 4.68) for preterm low birth weight. While these findings suggest a notable impact of periodontal disease on pregnancy complications, the presence of confounding factors limits full validation of the results. Furthermore, despite the strong association between periodontitis and miscarriage, no direct correlation was identified between miscarriage and specific periodontopathogenic bacteria such as Porphyromonas gingivalis, Tannerella forsythia, and Fusobacterium nucleatum [22]. A correlation analysis further elucidated the relationships between blood parameters and oral indices, highlighting potential links between systemic inflammation and periodontal health. In the case group, a significant positive correlation was observed between PCT and the GI (r = 0.3196, *p* = 0.0417), suggesting that elevated inflammatory markers may be associated with worsening periodontal health. Conversely, in the control group, a significant negative correlation was found between the PLR and CAL (r = −0.3515, *p* = 0.0242), indicating that better inflammatory regulation may contribute to healthier periodontal status in females without a history of miscarriage. Despite these significant correlations, no other notable relationships were detected among the remaining parameters, highlighting the complex interplay between systemic inflammation, periodontal health, and reproductive outcomes, thereby warranting further investigation. Existing evidence suggests that periodontitis may contribute to systemic comorbidities by inducing low-grade chronic inflammation, with individuals suffering from severe periodontitis exhibiting elevated levels of proinflammatory mediators such as interleukin-1 (IL-1), interleukin-6 (IL-6), C-reactive protein (CRP), and fibrinogen, along with increased neutrophil counts, compared with healthy controls [23,24]. Moreover, a recent review analyzing studies on inflammation indices in periodontal diseases underscored a strong association between periodontitis and systemic inflammatory markers, including PCT and the NLR, PLR, lymphocyte-to-monocyte ratio (LMR), delta neutrophil index (DNI), and sSII. However, the available evidence regarding the red blood cell distribution width (RDW) and PDW remains limited. Additionally, the review highlighted methodological limitations in the included studies and suggested that a single blood index may not be sufficient for predicting severe periodontitis [25]. In this study, the RDW, PDW, LMR, DNI, and SII were not evaluated; instead, we focused on PCT and the PLT, MPV/PLT, MLR, PLR, NLR, WBC, MPV, and NC. Notably, the PLR and PCT were found to be associated with periodontitis, with PCT showing a positive correlation with the GI in the case group, possibly reflecting the impact of active inflammation and vascular response. Conversely, the PLR demonstrated a negative correlation with CAL in the control group, suggesting a potential link between periodontal tissues and chronic systemic inflammation. These findings highlight how different blood parameters may reflect distinct inflammatory responses in the acute and chronic phases of periodontitis, reinforcing the need for further research to elucidate their role in systemic inflammation and periodontal disease progression. Hierarchical binary logistic regression analyses identified CAL as the strongest predictor of miscarriage risk (*p* < 0.0001), reinforcing the critical role of periodontal health in reproductive outcomes.

In a related study, Li et al. [26] identified several significant risk factors for spontaneous abortion, including advanced maternal age, a history of embryonic arrest, thyroid dysfunction, polycystic ovary syndrome, the use of assisted reproductive technologies, environmental exposures, depression, and stress. Supporting these findings from an inflammatory perspective, a prospective, controlled study conducted by Taşkomur and colleagues compared serum procalcitonin levels, NLRs, and PLRs among groups with threatened abortion, spontaneous abortion, and healthy pregnancies; procalcitonin levels and NLRs were found to be significantly higher in the threatened abortion group. ROC analyses demonstrated that both procalcitonin and the NLR had statistically significant diagnostic value in distinguishing this clinical condition [27].

CAL is not just a measure of periodontitis but an independent predictor of miscarriage risk, possibly due to systemic inflammation and immune dysregulation. While other blood parameters provided some insights into risk stratification, their discriminative power was comparatively weaker, underscoring the importance of periodontal health as a reliable marker. These results highlight the potential benefits of integrating periodontal assessments into obstetric care to enhance early detection and intervention strategies. However, further research is necessary to fully elucidate the systemic effects of periodontal inflammation on miscarriage and its long-term reproductive consequences. The strengths of this study include the use of CBC tests for evaluating inflammatory markers, providing a reliable and non-invasive method with significant advantages. Oral examinations were conducted by two independent periodontologists, and the measurement reliability was confirmed through an ICC analysis, enhancing the accuracy of the obtained data. Additionally, blood parameters were analyzed using standardized laboratory methods, ensuring the reliability of the biochemical variables. This study has several limitations. The intraoral examinations were performed at the patient’s bedside and relied solely on a clinical evaluation, which may have limited their diagnostic sensitivity. This approach could lead to less precise results compared with advanced diagnostic methods such as radiographic imaging or molecular analyses. Furthermore, as this study was conducted at a single center, its findings may not be widely generalizable, and the influence of geographical, cultural, or socioeconomic factors could not be fully evaluated. Furthermore, a detailed examination of systemic inflammatory markers (e.g., cytokines) or specific periodontal pathogens, which could have provided deeper insights into the underlying mechanisms, was not conducted. Future studies should incorporate advanced diagnostic methods, including radiographic imaging, molecular biological analyses, and examinations of specific inflammatory biomarkers. Prospective multicenter studies with larger sample sizes could improve the generalizability of the findings.

## 5. Conclusions

Considering the limitations of this study, CAL, a key indicator of periodontal disease severity, was found to be significantly associated with miscarriage and may serve as a potential marker for identifying individuals at risk. The elevated PCT levels observed in women who experienced miscarriage, along with their positive correlation with gingival inflammation, suggest a potential systemic inflammatory link between periodontal health and pregnancy outcomes.

## Figures and Tables

**Table 1:** Blood counts of study subjects and the relationship between miscarriages and controls.

Blood Counts	Miscarriages, N = 41 (50%)	Controls, N = 41 (50%)	*p*-Value
PLT	267,804.87 (267,000)	228,853.66 (225,000)	**0.004 ^1^**
MPV/PLT	0.04 (0.04)	0.05 (0.05)	**0.001 ^1^**
MLR	148.08 (130)	117.12 (106.52)	**0.004 ^1^**
PLR	0.28 (0.24)	0.33 (0.33)	0.919 ^1^
NLR	3.58 (2.81)	3.95 (3.47)	**0.000 ^1^**
WBC	8519.02 (7930)	10,301.71 (9650)	**0.002 ^1^**
MPV	12.07 (11.8)	13.39 (12.8)	**0.031 ^1^**
PCT	0.28 (0.27)	0.25 (0.25)	**0.017 ^1^**
NC	5.99 (5.47)	7.54 (6.99)	**0.000 ^1^**

Mean (Median); ^1^ Mann–Whitney U test. Values highlighted in bold represent data with a *p*-value less than 0.05, indicating statistical significance. PLT: Platelet Count; MPV/PLT: Mean Platelet Volume-to-Platelet Ratio; MLR: Monocyte-to-Lymphocyte Ratio; PLR: Platelet-to-Lymphocyte Ratio; NLR: Neutrophil-to-Lymphocyte Ratio; WBC: White Blood Cell Count; MPV: Mean Platelet Volume; PCT: Plateletcrit; NC: Neutrophil Count.

**Table 2:** Oral indices of subjects and the relationship between miscarriages and controls.

Oral Indices	Miscarriages, N = 41 (50%)	Controls, N = 41 (50%)	*p*-Value
Pocket Depth	2.00 (1.99)	1.91 (1.9)	0.264 ^1^
Clinical Attachment Loss	2.02 (1.99)	0.71 (0)	**<0.001** ^1^
Gingival Index	0.89 (0.88)	1.00 (1.07)	0.358 ^1^
Periodontal Index	1.05 (1.05)	4.02 (1.08)	0.937 ^1^
Simplified Calculus Index	0.49 (0.46)	0.47 (0.48)	0.812 ^1^

Mean (Median); ^1^: Mann–Whitney U test. Values highlighted in bold represent data with a *p*-value less than 0.05, indicating statistical significance.

**Table 3:** Spearman’s correlation analysis comparing blood counts and oral health indices in patients who suffered miscarriage.

	Pocket Depth	CAL	GI	PI	SCI
PLT	0.1868 (*p* = 0.242)	0.2098 (*p* = 0.188)	0.2553 (*p* = 0.107)	0.1717 (*p* = 0.283)	0.1647 (*p* = 0.303)
MPV/PLT	−0.262 (*p* = 0.098)	−0.2831 (*p* = 0.072)	−0.1975 (*p* = 0.215)	−0.144 (*p* = 0.369)	−0.1306 (*p* = 0.415)
MLR	−0.0357 (*p* = 0.824)	−0.0281 (*p* = 0.861)	0.1417 (*p* = 0.376)	0.0968 (*p* = 0.547)	0.0291 (*p* = 0.856)
PLR	−0.0671 (*p* = 0.676)	−0.0812 (*p* = 0.613)	−0.1798 (*p* = 0.260)	−0.1024 (*p* = 0.524)	−0.1467 (*p* = 0.360)
NLR	−0.0552 (*p* = 0.731)	−0.0599 (*p* = 0.710)	0.053 (*p* = 0.742)	0.0366 (*p* = 0.820)	−0.0517 (*p* = 0.748)
WBC	0.0484 (*p* = 0.763)	0.0371 (*p* = 0.817)	−0.1132 (*p* = 0.480)	−0.0395 (*p* = 0.806)	−0.0695 (*p* = 0.666)
MPV	−0.1511 (*p* = 0.345)	−0.1611 (*p* = 0.314)	0.0238 (*p* = 0.882)	0.0103 (*p* = 0.949)	0.0102 (*p* = 0.949)
PCT	0.1102 (*p* = 0.492)	0.1359 (*p* = 0.397)	**0.3196 (*p* = 0.041)**	0.2122 (*p* = 0.182)	0.2087 (*p* = 0.190)
NC	0.0946 (*p* = 0.556)	0.0819 (*p* = 0.610)	−0.0097 (*p* = 0.952)	0.0368 (*p* = 0.819)	−0.0199 (*p* = 0.901)

Values highlighted in bold represent data with a *p*-value less than 0.05, indicating statistical significance; CAL: Clinical Attachment Loss; GI: Gingival Index; PI: Periodontal Index; SCI: Simplified Calculus Index; PLT: Platelet Count; MPV/PLT: Mean Platelet Volume-to-Platelet Ratio; MLR: Monocyte-to-Lymphocyte Ratio; PLR: Platelet-to-Lymphocyte Ratio; NLR: Neutrophil-to-Lymphocyte Ratio; WBC: White Blood Cell Count; MPV: Mean Platelet Volume; PCT: Plateletcrit; NC: Neutrophil Count.

**Table 4:** Spearman’s correlation analysis comparing blood counts and oral health indices in control patients.

	Pocket Depth	CAL	GI	PI	SCI
PLT	0.1781 (*p* = 0.265)	0.1423 (*p* = 0.374)	−0.0537 (*p* = 0.739)	0.0577 (*p* = 0.719)	0.0172 (*p* = 0.914)
MPV/PLT	−0.1084 (*p* = 0.499)	−0.1478 (*p* = 0.356)	0.0519 (*p* = 0.747)	−0.0596 (*p* = 0.711)	0.0182 (*p* = 0.910)
MLR	0.1071 (*p* = 0.505)	0.1812 (*p* = 0.256)	0.1247 (*p* = 0.437)	0.1609 (*p* = 0.314)	−0.1299 (*p* = 0.418)
PLR	−0.0164 (*p* = 0.919)	**−0.3515 (*p* = 0.024)**	0.0421 (*p* = 0.793)	−0.0440 (*p* = 0.784)	−0.0891 (*p* = 0.579)
NLR	−0.0210 (*p* = 0.896)	−0.0714 (*p* = 0.657)	0.0820 (*p* = 0.610)	−0.0003 (*p* = 0.998)	0.0439 (*p* = 0.785)
WBC	0.0488 (*p* = 0.761)	−0.1179 (*p* = 0.462)	0.1022 (*p* = 0.525)	0.0394 (*p* = 0.806)	0.1742 (*p* = 0.276)
MPV	0.1688 (*p* = 0.291)	−0.1700 (*p* = 0.288)	0.0533 (*p* = 0.740)	0.0363 (*p* = 0.821)	0.2195 (*p* = 0.167)
PCT	0.2799 (*p* = 0.076)	0.1002 (*p* = 0.533)	−0.0075 (*p* = 0.962)	0.1082 (*p* = 0.500)	0.1039 (*p* = 0.518)
NC	0.0585 (*p* = 0.716)	−0.0688 (*p* = 0.668)	0.1438 (*p* = 0.369)	0.0587 (*p* = 0.715)	0.3500 (*p* = 0.400)

Values highlighted in bold represent data with a *p*-value less than 0.05, indicating statistical significance. CAL: Clinical Attachment Loss; GI: Gingival Index; PI: Periodontal Index; SCI: Simplified Calculus Index; PLT: Platelet Count; MPV/PLT: Mean Platelet Volume-to-Platelet Ratio; MLR: Monocyte-to-Lymphocyte Ratio; PLR: Platelet-to-Lymphocyte Ratio; NLR: Neutrophil-to-Lymphocyte Ratio; WBC: White Blood Cell Count; MPV: Mean Platelet Volume; PCT: Plateletcrit; NC: Neutrophil Count.

**Table 5:** Presentation of hierarchical binomial logistic regression analysis results.

Predictor	Estimate	SE	Z	*p*-Value	Odds Ratio	Lower (95% Cl)	Upper (95% Cl)	Deviance	AIC	R^2^_MCF_
Model 1										
Intercept ᵃ	4.4858	1.2069	3.7168	0.000	88.7478	8.3335	945.125	60.18404134	64.18404134	0.470565744
CAL	2.9251	0.6767	4.3226	0.000	0.0537	0.0142	0.2021			
Model 2										
Intercept ᵃ	50.3424	28.8058	1.7476	0.081	7.30157 × 10^21^	0.0022	2.41783 × 10^46^	52.82038093	84.82038093	0.535343283
PLT	−0.0003	0.0001	−2.6313	0.009	0.9997	0.9995	0.9999			
MPV/PLT	72.165	122.7056	0.5881	0.557	2.19219 × 10^31^	0	6.1656 × 10^135^			
MLR	0.0644	0.0399	1.6155	0.106	1.0665	0.9864	1.1532			
PLR	9.2487	12.5895	0.7346	0.463	10,390.8718	0	5.40817 × 10^14^			
NLR	−3.124	1.4929	−2.0926	0.036	0.044	0.0024	0.8204			
WBC	0.0002	0.0007	0.3349	0.738	1.0002	0.9989	1.0016			
MPV	0.0474	0.5071	0.0935	0.926	1.0486	0.3881	2.8332			
PCT	200.4991	94.0648	2.1315	0.033	1.19029 × 10^87^	10,141,534.58	1.397 × 10^167^			
NC	2.354	1.1638	2.0226	0.043	10.5272	1.0756	103.0353			

ᵃ Ref: Represents reference level; SE: Standard Error; AIC: Akaike Information Criterion; R^2^_MCF_: McFadden’s R^2^; CAL: Clinical Attachment Loss; PLT: Platelet Count; MPV/PLT: Mean Platelet Volume-to-Platelet Ratio; MLR: Monocyte-to-Lymphocyte Ratio; PLR: Platelet-to-Lymphocyte Ratio; NLR: Neutrophil-to-Lymphocyte Ratio; WBC: White Blood Cell Count; MPV: Mean Platelet Volume; PCT: Plateletcrit; NC: Neutrophil Count.

**Table 6:** ROC analysis results with Youden’s Index.

Predictor	AUC	Youden’s Index	Optimal Threshold	Sensitivity	Specificity
CAL	0.8691	0.7561	1.4	1.0000	0.7561
PLT	0.6871	0.3415	250,000	0.6341	0.7073
NLR	0.2885	0.0732	11.3684	0.0732	1.00000
PCT	0.6523	0.2439	0.23	0.8537	0.3902
NC	0.2823	0.0000	15.6400	0.0000	1.0000

AUC: Area Under the Curve; CAL: Clinical Attachment Loss; PLT: Platelet Count; NLR: Neutrophil-to-Lymphocyte Ratio; PCT: Plateletcrit; NC: Neutrophil Count.

## Data Availability

The data supporting the findings of this study are available from the corresponding author upon reasonable request.

## Abbreviations

The following abbreviations are used in this manuscript:

PI	Plaque Index
GI	Gingival Index
PD	Probing Depth
CAL	Clinical Attachment Loss
SCI	Simplified Calculus Index
CRP	C-reactive protein
IL-1	Interleukin-1
IL-6	Interleukin-6
PLR	Platelet-to-Lymphocyte Ratio
MLR	Monocyte-to-Lymphocyte Ratio
NLR	Neutrophil-to-Lymphocyte Ratio
ENR	Eosinophil-to-Neutrophil Ratio
ELR	Eosinophil-to-Lymphocyte Ratio
PLT	Platelet Count
MPV/PLT	Mean Platelet Volume-to-Platelet Ratio
DNI	Delta neutrophil index
SII	Systemic immune-inflammation index
WBC	White Blood Cell Count
RDW	Red cell distribution width
PDW	Platelet distribution width
MPV	Mean Platelet Volume
PCT	Plateletcrit
NC	Neutrophil Count
CBC	Complete blood count
ICC	Intraclass correlation coefficient
LMP	Last menstrual period
